# Cholecystectomy with jejunoileal bypass ameliorates diabetic metabolism in mice with type 2 diabetes through modulation of FXR and TGR5 signaling

**DOI:** 10.1016/j.metop.2025.100437

**Published:** 2025-12-22

**Authors:** Haixin Yin, Weijie Chen, Xiaodong He, JianPing Zeng

**Affiliations:** a Hepato-pancreato-biliary Center, Beijing Tsinghua Changgung Hospital, School of Clinical Medicine, Tsinghua University, Beijing, 102218, PR China; b Department of General Surgery, Peking Union Medical College Hospital, Chinese Academy of Medical Sciences & Peking Union Medical College, Shuaifuyuan 1#, Dongcheng District, Beijing, 100730, PR China

**Keywords:** T2DM, Glucose, Lipid, Biliary diversion, Bile acids

## Abstract

**Objectives:**

Our aim was to investigate the improvement in glucose and lipid profiles and the mechanism involving bile acid receptors FXR and TGR5 following cholecystectomy with jejunoileal bypass(CJB) in mice with type 2 diabetes mellitus (T2DM).

**Methods:**

Twenty male mice with T2DM were randomly assigned to CJB and sham groups. We analyzed fasting blood glucose (FBG), insulin, total cholesterol (TC), triglycerides (TG), high-density lipoprotein cholesterol (HDL-C), low-density lipoprotein cholesterol (LDL-C), free fatty acids (FFA), glucagon-like peptide-1 (GLP-1), gastric inhibitory polypeptide (GIP), and total bile acids (TBA) levels. Expression levels of the farnesoid X receptor (FXR) and G protein-coupled bile acid receptor (TGR5) in liver, muscle, and adipose tissue were assessed post-sacrifice.

**Results:**

Despite pair-feeding, the CJB group exhibited lower body weights than the sham group at 24 weeks post-surgery (*P* = 0.004). The levels of FBG (*P* < 0.001) and insulin (*P* = 0.013) in the CJB group significantly decreased compared to the baseline. The intraperitoneal glucose and insulin tolerance tests demonstrated improved glucose tolerance (*P* = 0.004) and insulin sensitivity (*P* = 0.001). GLP-1 levels significantly increased (*P* = 0.002). The levels of TC (*P* < 0.001) and LDL-C (*P* < 0.001) were decreased, while the levels of TG (*P* = 0.003), HDL-C (*P* < 0.001), and FFA (*P* < 0.001) were increased at the 24th postoperative week. Furthermore, TBA concentrations were higher in the CJB group than in the control group (*P* < 0.001). The expression of FXR in the liver (*P* = 0.034), muscle (*P* = 0.003) and adipose tissue (*P* = 0.045) and that of TGR5 in the liver (*P* = 0.024), muscle (*P* = 0.026) and adipose tissue (*P* = 0.004) were upregulated after surgery.

**Conclusions:**

In mice with T2DM, cholecystectomy with jejunoileal bypass modulates BA–FXR/TGR5 signaling and is associated with metabolic improvement.

## 1 Introduction

Cholecystectomy is the most commonly performed surgical procedure for patients with cholelithiasis and polyps [1,2]. As a storage organ for bile, its removal leads to the continuous secretion of bile into the intestine. Growing evidence suggests that cholecystectomy itself alters the physiological cycling pattern of bile acids, such as increased serum bile acid levels and accelerated enterohepatic circulation [[3], [4], [5]], thereby triggering a series of complex metabolic changes [5,6], with both positive and negative effects being reported [7]. The beneficial metabolic signaling of bile might be amplified by further surgically altering its spatial distribution and actively diverting it to intestinal regions critical for metabolic regulation. Accordingly, this study designed a novel combined surgical procedure: cholecystectomy with side-to-side jejunoileal bypass. It allows bile and chyme to bypass most of the small intestine, entering the distal ileum and colon directly and early, thereby exposing these intestinal segments to stimulation by high concentrations of bile.

The distal ileum and colon are precisely the “key regions” within the gut that sense nutrient and chemical signals and secrete various hormones to regulate systemic metabolism. Enteroendocrine L cells, which are highly enriched in these areas, play a central role. Upon effective stimulation, L cells secrete several incretins, including Glucagon-like peptide-1 (GLP-1), which play vital roles in regulating blood glucose, improving insulin resistance, and modulating lipid metabolism. How, then, are these L cells activated? Previous research has suggested that intestinal neural signals, nutrients, and hormones can activate L cells [[8], [9], [10]]. However, the roles of specific receptors for bile acid signaling [11] -the Farnesoid X receptor (FXR) and the G protein-coupled bile acid receptor (TGR5)-have been scarcely investigated in this context.

Studies have shown that biliary intervention has beneficial effects on patients with dyslipidemia but not in patients with normal lipid metabolism [12,13], indicating different baseline metabolic profiles have different responses to the same intervention. Therefore, individuals with type 2 diabetes mellitus (T2DM), characterized by numerous metabolic disorders, may show completely different outcomes after cholecystectomy from those of patients with normal metabolisms. Normally, the levels of glucose and lipids are exquisitely regulated, and compensation mechanisms make some disturbances invisible. However, fine stabilization control is impaired in T2DM, and the positive or negative effects of disturbances in glucose and lipid metabolism might be exposed. The *db/db* mice are widely used T2DM model showing hyperglycemia and insulin resistance very similar to those in T2DM patients due to a mutation of the leptin receptor [14]. Therefore, we performed this study to investigate the changes in glucose and lipid metabolism after cholecystectomy with jejunoileal bypass (CJB) in *db/db* mice by examining the associated changes in bile acid flow and the activation of ileal/colonic FXR, TGR5.

## 2 Materials and methods

### 2.1 Animals

The feeding process of mice and some of the methods mentioned later were performed as previously described [15]. Eight-week-old male *db/db* mice with similar body weights and fasting blood glucose (FBG) levels were purchased (National Rodent Laboratory Animal Resources, Shanghai, China), female mice were excluded in order to minimize the metabolic impact of menstrual cycles. Mice were housed individually with a 12-h light/dark cycle (lights off at 10:00 a.m.) in a climate-controlled environment (18–22 °C temperature and 50 % humidity) in the Animal Center of our hospital. All mice had free access to tap water, and they were fed a standard chow diet before the operations. After 1 week of acclimation, twenty mice were randomly divided into the CJB group and the sham group, and CJB or sham surgery was performed, respectively. All experiments and surgical procedures were carried out in accordance with the Laboratory Animal Ethics Committee of our hospital.

### 2.2 Surgeries and postoperative care

**Fig. 1:**
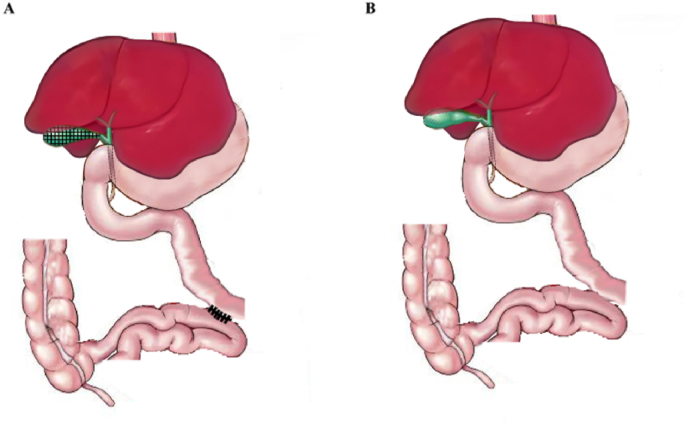
Sketch image of cholecystectomy with jejunoileal bypass and sham surgery. A, The sketch of cholecystectomy with side-to-side jejunoileal bypass. The gallbladder was removed (cholecystectomy). A side-to-side anastomosis was created between the proximal jejunum and the distal ileum, allowing a portion of bile to flow directly into the distal ileum. B, The sketch of sham surgery. It involved the same abdominal incision and intestinal manipulation without cholecystectomy or the jejunoileal anastomosis.

The mice were fasted overnight and anesthetized with isoflurane. The abdominal skin was wiped with 75 % alcohol, and the abdominal cavity was accessed through a small medial laparotomy. The procedure was performed using a 15 × surgical microscope. The cystic duct was ligated, and the gallbladder was gently removed. A side-to-side anastomosis was performed by continuous suture using 9-0 needles with thread between the jejunum 5 cm distal to the ligament of Treitz and the ileum 5 cm proximal to the ileocecal valve, and the anastomosis was 2–3 mm long. Abdominal wall was closed with 5-0 nylon suture that was removed 7 days after the procedure. The sham operation involved the same surgical incisions but omitted the intestinal anastomosis and cholecystectomy (Fig. 1). All mice received analgesia postoperatively and resumed a chow diet the day after surgery. The operative mortality was 50 %–70 % in the preliminary experiments, mostly due to excessive anesthesia, but the mortality greatly decreased to nearly 5 % with increased experience. In the present study, all twenty mice survived after surgery, and there were no complications until the mice were sacrificed by cervical dislocation. Mouse survival longer than 1 week without complications was defined as a successful operation. The procedures were well tolerated, as indicated by normal body weight gain during the observational period.

### 2.3 Food intake and body weight

All mice were housed individually and fed the same amount of chow (pair-fed) [16] to reduce the potential impact of food intake on lipid and glucose metabolism. The mice were given a normal diet (5 % fat rat chow diet, Keao Xieli, China) and had free access to water. The maximum 24-h food intake and body weight were measured weekly for the first month and every two weeks thereafter. Body weight was measured after overnight fasting (10 h); then, the mice had free access to chow for one day, and the maximum 24-h food intake was measured in the two groups. After that, the intake was calculated by subtracting the weight of the remaining food from the weight of the food provided. The mice were pair-fed thereafter.

### 2.4 Intraperitoneal glucose tolerance test (IPGTT) and intraperitoneal insulin tolerance test (IPITT)

The IPGTT was performed before surgery and at the 4th, 13th, and 23rd weeks postoperatively. After fasting overnight (10 h), all mice received 2 g/kg glucose by intraperitoneal injection, and blood glucose was measured 0, 30, 60, 90, and 120 min after glucose administration with a One Touch glucometer (Roche, Germany). The IPITT was performed before surgery, and at the 5th, 14th, and 24th weeks. The mice were fasted for 6 h and then administered 1.0 U/kg human insulin (Novo Nordisk, Denmark) intraperitoneally. Blood sampling was performed to measure blood glucose levels 0, 30, 60, 90, and 120 min after insulin injection. Insulin sensitivity was assessed by the IPITT combined with glucose and insulin levels [17].

### 2.5 Biochemical tests

Blood glucose was measured through the tail vein weekly for the first month and every two weeks thereafter with a One Touch glucometer. Blood samples were collected by piercing the submaxillary vein every two weeks for the first month and monthly thereafter. At the end of the study period, the mice were anesthetized with isoflurane, and blood samples were collected by eyeball extraction. After centrifugation at 3000 rpm at 4 °C for 15 min, the plasma samples were separated immediately and stored at −80 °C until further analysis. Enzyme-linked immunosorbent assay kits were used to measure insulin (Youersheng, China), glucagon-like peptide-1 (GLP-1) (Youersheng, China) and gastric inhibitory polypeptide (GIP) (Youersheng, China). The levels of total cholesterol (TC), triglycerides (TG), high-density lipoprotein cholesterol (HDL-C), low-density lipoprotein cholesterol (LDL-C), free fatty acids (FFA) and total bile acids (TBA) were measured with a fully automatic biochemical analyzer (Roche, Germany).

### 2.6 Western blotting

At the 24th postoperative week, all mice were sacrificed. FXR and TGR5 were extracted from fresh liver, distal ileum and colon thereby measured by Western blotting. Proteins were resolved by 10 % SDS-polyacrylamide gel electrophoresis and transferred onto 0.45 μm PVDF membranes. The membranes were blocked with blocking buffer for 2 h and probed with anti-FXR antibody (Cell Signaling Technology, USA) at a 1:5000 dilution, anti-TGR5 antibody (Abcam, UK) at a 1:10,000 dilution and anti-β-Actin (Cell Signaling Technology) at a 1:10,000 dilution overnight at 4 °C. After being washed with wash buffer three times, the membranes were incubated for 1 h at room temperature with goat anti-rabbit antibody (Zsgb-bio, China) at a 1:10,000 dilution. The relative protein concentrations were quantified by densitometry using the Versa Doc 1000 imaging system and Quantity One 4.4 software (Bio-Rad, Hercules, USA).

### 2.7 Statistical analysis

We conducted statistical analyses using SPSS Statistics software (version 24.0, IBM, USA) and drafted histograms using GraphPad software (version 7.0, GraphPad Prism, USA). Quantitative data are shown as the mean ± standard deviation (SD). Repeated measures ANOVA was used to compare the differences preoperatively and postoperatively, and multivariate analysis was used to compare the differences between the CJB and sham groups. *P* values less than 0.05 were considered statistically significant.

## 3 Results

### 3.1 Food intake and body weight

**Fig. 2:**
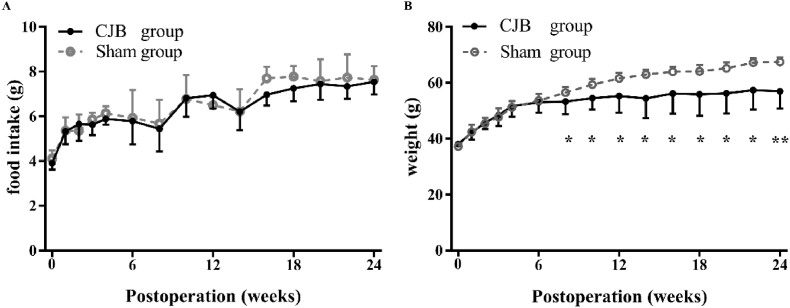
The maximum 24-h food intake and weights of mice in the CJB and sham groups. A, The maximum 24-h food intake of mice in the two groups. There was no significant difference between the groups. B, Body weights of mice in the two groups. The body weights of mice in the CJB group were less than those of mice in the sham group beginning in the 8th postoperative week. Eight-week-old male *db/db* mice, CJB: n = 10, Sham: n = 10. CJB, cholecystectomy with jejunoileal bypass. The data are presented as the mean ± SD, *P < 0.05, **P < 0.01 compared with the sham group.

The maximum 24-h food intake (*P* = 0.30) or body weight (*P* = 0.17) between two groups were similar before surgery, and also no significant differences after surgery. However, the weights in the CJB group were lower than those in the sham group beginning in the 8th week after surgery. At the 24th postoperative week, the weight in the sham group was 67.5 ± 1.6 g and that in the CJB group was 56.9 ± 6.1 g (*P* = 0.004, Fig. 2).

### 3.2 Effects on fasting blood glucose

**Fig. 3:**
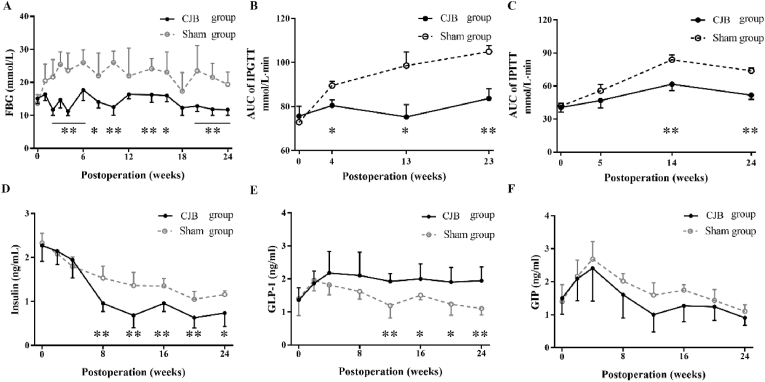
Glucose changes after CJB in diabetic mice. A, The fasting blood glucose curve showing significantly decreased fasting blood glucose in the CJB group beginning in the 2nd postoperative week. B, Changes in the AUC of the IPGTT. C, Changes in the AUC of the IPITT. D, The insulin level curve showing that the insulin level decreased significantly in the CJB group beginning in 8th postoperative week. E, The GLP-1 level curve showing that the GLP-1 level increased significantly in the CJB group beginning in the 12th postoperative week. F, The GIP level curve showing that there were no significant differences in the GIP level before or after surgery. Eight-week-old male *db/db* mice, CJB: n = 10, Sham: n = 10. CJB, cholecystectomy with jejunoileal bypass; FBG, fasting blood glucose; AUC, area under the curve; IPGTT, intraperitoneal glucose tolerance test; IPITT, intraperitoneal insulin tolerance test; GLP-1, glucagon-like peptide-1; GIP, gastric inhibitory polypeptide. The data are presented as the mean ± SD, *P < 0.05, **P < 0.01.

There were no significant differences in FBG between the two groups before surgery (CJB: 15.1 ± 1.5 mmol/L *vs*. sham: 13.7 ± 2.6 mmol/L, *P* = 0.25). At the 24th postoperative week, the FBG in the CJB group decreased to 11.7 ± 1.8 mmol/L compared to the baseline level (*P* = 0.012) and was lower than that of the sham group (19.4 ± 3.7 mmol/L, *P* < 0.001 Fig. 3A).

### 3.3 Changes in glucose tolerance and insulin sensitivity

There was no significant difference in the area under the curve (AUC) of the IPGTT before surgery (CJB: 75.7 ± 4.5 mg/dL·min *vs*. sham: 72.9 ± 2.7 mg/dL·min, *P* = 0.63). The CJB group had significant improved clearance of intraperitoneal injected glucose compared to that of the sham group. The AUC of the IPGTT in the CJB group (83.7 ± 4.5 mg/dL·min) was not significantly different from that of the baseline (*P* = 0.22) but was significantly lower than that of the sham group (104.9 ± 2.7 mg/dL·min, *P* = 0.004) at the 23rd postoperative week (Fig. 3B).

There was also no significant difference in the AUC of the IPITT before surgery (CJB: 40.6 ± 4.4 mg/dL·min *vs*. sham: 41.8 ± 2.5 mg/dL·min, *P* = 0.82). Insulin sensitivity improved after CJB. At the 24th postoperative week, the AUC of the IPITT in the CJB group (51.6 ± 3.9 mg/dL·min) was similar to the baseline (*P* = 0.08) but was significantly lower than that of the sham group (73.8 ± 2.5 mg/dL·min, *P* = 0.001) (Fig. 3C).

### 3.4 Biochemical tests

There was no significant difference in the insulin level before surgery (CJB: 2.3 ± 0.4 ng/mL *vs*. sham: 2.3 ± 0.2 ng/mL, *P* = 0.76). After surgery, the insulin level in the CJB group decreased at the 8th postoperative week and decreased to 0.7 ± 0.3 ng/mL at the 24th postoperative week (*P* < 0.001), which was also lower than that in the sham group (1.2 ± 0.1 ng/mL, *P* = 0.013, Fig. 3D). Similarly, the level of GLP-1 before surgery was not different between the two groups (CJB: 1.4 ± 0.4 ng/mL *vs*. sham: 1.4 ± 0.5 ng/mL, *P* = 0.88). After surgery, the GLP-1 level increased at the 12th postoperative week in the CJB group and increased to 1.9 ± 0.4 ng/mL at the 24th postoperative week (*P* = 0.04) compared to baseline, which was also higher than that in the sham group (1.1 ± 0.2 ng/mL, *P* = 0.002, Fig. 3E). There were no significant differences in the level of GIP before surgery (CJB: 1.5 ± 0.5 ng/mL *vs*. sham: 1.4 ± 0.5 ng/mL, *P* = 0.73) or at the 24th postoperative week (CJB: 0.9 ± 0.2 ng/mL *vs*. sham: 1.1 ± 0.2 ng/mL *P* = 0.15, Fig. 3F).

### 3.5 Effects on lipid metabolism

**Fig. 4:**
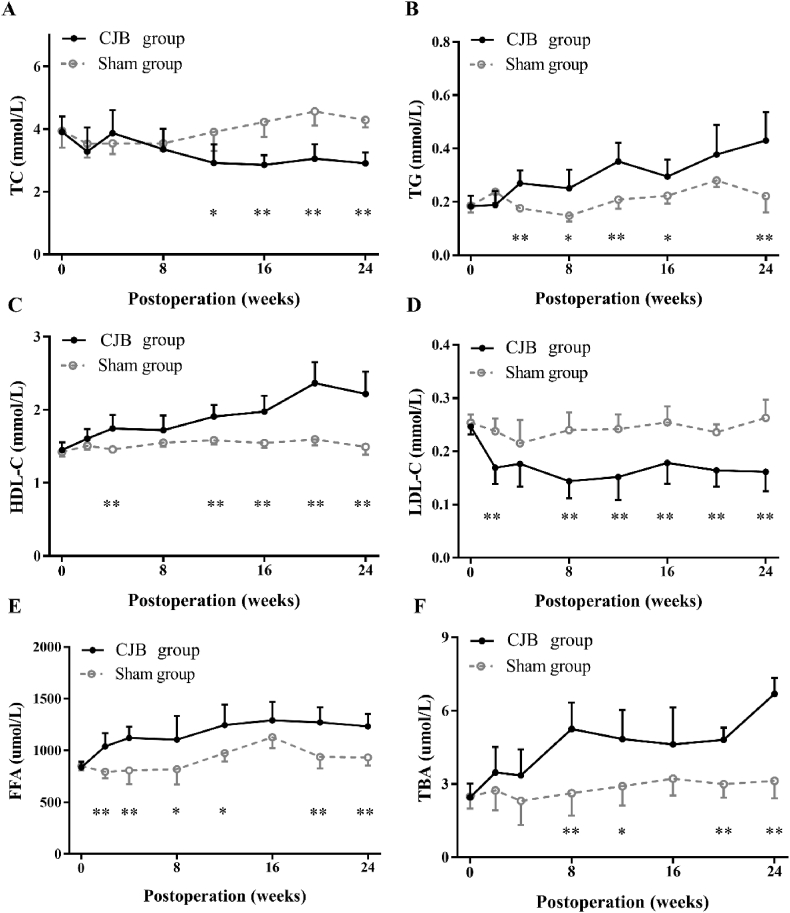
Lipid changes after CJB and the level of total bile acids in serum. A, The TC curve showing that the TC level decreased significantly beginning in the 12th postoperative week. B, The TG curve showing that the TG level increased beginning in the 4th postoperative week, except for the 20th week. C, The HDL-C curve showing that the HDL-C level increased beginning in the 4th postoperative week, except for the 8th week. D, The LDL-C curve showing that the LDL-C level decreased beginning in the 2nd postoperative week, except for the 4th week. E, The FFA curve showing that the FFA level increased beginning in the 2nd postoperative week, except for the 16th week. F, The TBA curve showing that the TBA level increased beginning in the 8th postoperative week, except for the 16th week. Eight-week-old male *db/db* mice, CJB: n = 10, Sham: n = 10. CJB, cholecystectomy with jejunoileal bypass; TC, total cholesterol; TG, triglycerides; HDL-C, high-density lipoprotein cholesterol; LDL-C, low-density lipoprotein cholesterol; FFA, free fatty acids; TBA, total bile acids. The data are presented as the mean ± SD, *P < 0.05, **P < 0.01.

Before surgery, there were no significant differences in the levels of TC (*P* = 0.93), TG (*P* = 0.92), HDL-C (*P* = 0.61), LDL-C (*P* = 0.93), FFA (*P* = 0.68) and TBA (*P* = 0.93). After CJB, the TC level decreased from 3.9 ± 0.3 mmol/L to 2.9 ± 0.3 mmol/L (*P* = 0.003, Fig. 4A) and was lower than that of the sham group at the 24th postoperative week (CJB: 2.9 ± 0.3 mmol/L *vs*. sham: 4.3 ± 0.2 mmol/L, *P* < 0.001). The LDL-C level also decreased from 0.25 ± 0.01 mmol/L to 0.16 ± 0.04 mmol/L (*P* < 0.001) and was lower than that of the sham group (0.26 ± 0.03 mmol/L, *P* < 0.001) (Fig. 4D).

TG and HDL-C concentrations in the CJB group increased beginning in the 4th week after surgery. The TG level increased from 0.18 ± 0.04 mmol/l before surgery to 0.43 ± 0.10 mmol/l at the 24th postoperative week (*P* = 0.002) and was higher than that of the sham group (0.22 ± 0.06 mmol/L, *P* = 0.003, Fig. 4B). The HDL-C level increased from 1.5 ± 0.1 mmol/L to 2.2 ± 0.3 mmol/L (*P* = 0.001) and was higher than that of the sham group at the 24th postoperative week (1.5 ± 0.1 mmol/L, *P* < 0.001) (Fig. 4C). The FFA level increased from 837.0 ± 53.1 μmol/L to 1233.2 ± 120.6 μmol/L (*P* < 0.001) and was higher than that of the sham group at the 24th postoperative week (930.0 ± 78.8 μmol/L *P* < 0.001, Fig. 4E).

### 3.6 TBA and the expression of FXR and TGR5

The TBA concentration increased significantly beginning in the 8th postoperative week after CJB from 2.5 ± 0.6 μmol/L before surgery to 6.7 ± 0.7 μmol/L at the 24th postoperative week (*P* < 0.001) and was higher than that of the sham group (3.1 ± 0.7 μmol/L, *P* < 0.001) (Fig. 4F).

**Fig. 5:**
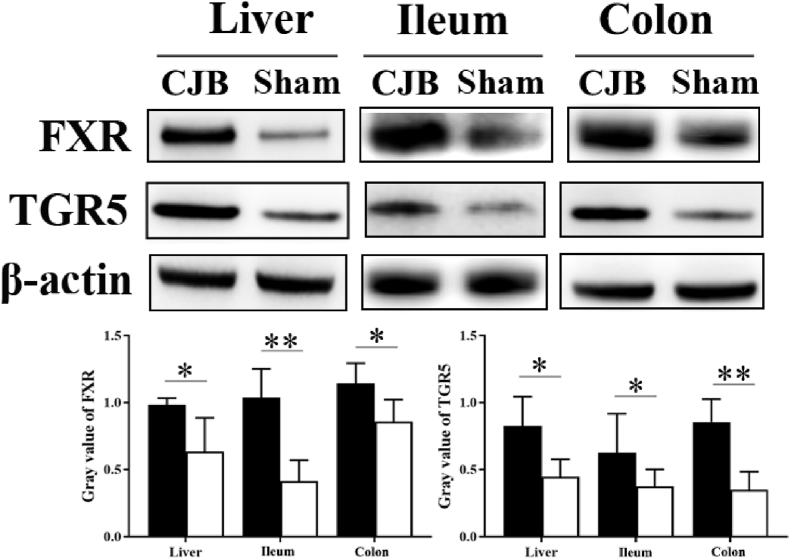
Expression of FXR and TGR5 in the liver, ileum and colon tissue. Samples were dissected and obtained from fresh liver, distal ileum and colon. Western blot analysis of FXR and TGR5 (Top), and the relative expression levels of FXR and TGR5 were increased after CJB. The gray values of FXR and TGR5 expression are also shown (bottom). Eight-week-old male *db/db* mice, CJB: n = 10, Sham: n = 10. CJB, cholecystectomy with jejunoileal bypass; FXR, farnesoid X receptor; TGR5, G protein-coupled bile acid receptor. The black bar indicates the CJB group; the white bar indicates the sham group, *P < 0.05, **P < 0.01.

The expression of FXR and TGR5 in the liver, distal ileum, and colon was measured by Western blotting (Fig. 5). The relative expression level of liver FXR in the CJB group (1.0 ± 0.1) was higher than that in the sham group (0.6 ± 0.3, *P* = 0.034). The relative expression level of ileum FXR in the CJB group (1.0 ± 0.2) was higher than that in the sham group (0.4 ± 0.2,*P* = 0.003). The relative expression level of colon FXR in the CJB group (1.1 ± 0.2) was higher than that in the sham group (0.9 ± 0.2, *P* = 0.045).

Moreover, the relative expression level of liver TGR5 in the CJB group (0.8 ± 0.2) was higher than that in the sham group (0.4 ± 0.1, *P* = 0.024). The relative expression level of ileum TGR5 in the CJB group (0.6 ± 0.3) was higher than that in the sham group (0.4 ± 0.1,*P* = 0.026). The relative expression level of colon TGR5 in the CJB group (0.9 ± 0.2) was higher than that in the sham group (0.4 ± 0.1, *P* = 0.004).

## 4 Discussion

Studies have shown that the same intervention can have different outcomes in patients with varying metabolic baselines, and it seems to be beneficial for patients with metabolic disorders [13]. T2DM was a systematic metabolic disease, the metabolic changes after biliary diversion may completely different from that in patients without T2DM. Our results showed that the level of FBG decreased significantly two weeks after surgery, and the insulin level also dropped six weeks after surgery. These results indicated that peripheral insulin resistance might be improved. The IPGTT and IPITT also confirmed the improvement in glucose metabolism. The mice were pair-fed, and the effect of food intake was negligible. However, the TBA level was significantly higher in the CJB group than the sham group.

BAs are synthesized from cholesterol in the liver, stored in the gallbladder and secreted into the intestine when a meal is ingested. Several studies have demonstrated that TBA levels increase markedly [4,5] and circulate faster [5] after cholecystectomy. Because the rhythmic functions of the GB acting as a bile reservoir and contractile pump were missing, the continuous secretion of BAs into the duodenal lumen enterohepatic circulation were influenced, and as a result, BAs circulated more quickly. While cholecystectomy alters the temporal rhythm of bile acid secretion, the combined jejunoileal bypass procedure in this study enables an active intervention in its spatial distribution. This approach allows bile to bypass the majority of the small intestine, resulting in its direct and continuous delivery to the distal ileum and colon. This critical physiological alteration strategically channels high concentrations of bile acids to specific intestinal regions that are highly sensitive to metabolic regulation.

The distal ileum and colon are key regions for endocrine function in the gut, densely populated with enteroendocrine L cells [18]. Upon stimulation, these cells secrete hormones such as glucagon-like peptide-1 (GLP-1), which plays a vital role in promoting insulin secretion, suppressing glucagon release, delaying gastric emptying, and improving insulin resistance [10]. Our findings demonstrate that following CJB, FXR and TGR5 was significantly upregulated in ileal and colonic tissues. This indicates an enhanced sensitivity of the distal gut to bile acid signaling, likely representing an adaptive remodeling of the intestinal mucosa in response to the new biliary milieu. It further implies that bile acids and their receptor pathways participate critically in the metabolic improvements observed after CJB.

Based on these findings, we propose a coherent core mechanism: CJB establishes a localized microenvironment of high bile acid concentration in the distal intestine through anatomical diversion. This microenvironment induces the upregulation of FXR and TGR5 in L cells. The increased receptor levels amplify bile acid signaling, synergistically enhancing the synthesis and secretion of metabolically beneficial hormones such as GLP-1 via immediate TGR5 activation and sustained FXR-mediated transcription. Ultimately, these hormones enter the systemic circulation, mediating global improvements in glucose and lipid metabolism.

In addition to the core hypothesis involving L-cell stimulation in the distal gut, BA-FXR and TGR5 signaling pathways may contribute to metabolic improvements through other pathways. In our study, we observed the upregulation of both FXR and TGR5 in the liver. Since hepatic FXR activation regulates genes involved in gluconeogenesis and lipid synthesis [[19], [20], [21], [22]], while TGR5 activation influences systemic energy expenditure and insulin sensitivity [11,23,24]., these findings suggest that these receptors also act in peripheral metabolic tissues to fine-tune glucose and lipid homeostasis, thereby complementing the gut-mediated effects of cholecystectomy with jejunoileal bypass.

Diabetic dyslipidemia has received much attention in recent years, but the underlying mechanism is complex and unclear [[25], [26], [27]]. Our study demonstrated a selective improvement in lipid metabolism concomitant with the amelioration of glucose homeostasis following CJB. Specifically, we observed elevated HDL-C levels and significant reductions in TC and LDL-C, despite concurrent increases in TG and FFA. The partial alleviation of diabetic dyslipidemia may be attributed, at least in part, to the improvement in systemic insulin resistance [28,29], given insulin's pivotal role in regulating lipid production and secretion. Furthermore, as glucose and lipid metabolic pathways are intrinsically linked, improved glycemic control likely exerts beneficial effects on lipid profiles through multifaceted mechanisms. The paradoxical rise in TG and FFA, however, might indicate an underlying impairment in lipid storage or an alteration in adipose tissue partitioning [30], warranting further investigation.

This study has several limitations that should be considered. The causality between less weight gain and metabolism needed to be further studied. In particular, future studies employing cellular models and genetic or pharmacological modulation of the FXR/TGR5 pathway are warranted to establish direct mechanistic causality. In addition, the relatively short observation period also limits the evaluation of long-term efficacy and safety. Moreover, species differences may limit translating the findings of this study from mice to humans, but diabetic mice can more or less simulate the pathogenesis of T2DM in humans. In summary, CJB led to notable improvements in glucose and lipid metabolism, accompanied by elevated systemic bile acid levels in mice with T2DM. These results underscore the importance of bile acid-mediated signaling in metabolic regulation and open promising avenues for developing novel surgical and pharmacological strategies for T2DM management.

## 5 Conclusions

In mice with T2DM, cholecystectomy with jejunoileal bypass modulates BA–FXR/TGR5 signaling and is associated with metabolic improvement.

## CRediT authorship contribution statement

**Haixin Yin:** Writing – original draft, Software, Formal analysis, Data curation. **Weijie Chen:** Writing – review & editing. **Xiaodong He:** Funding acquisition. **JianPing Zeng:** Writing – review & editing, Conceptualization.

## Ethical approval

The study was approved by the Ethics Committee of PUMCH at the Chinese Academy of Medical Sciences and Peking Union Medical College. All the experimental procedures were conform to the guidelines from Directive 2010/63/EU of the European Parliament on the protection of animals used for scientific purposes.

## Availability of data and materials

The datasets generated and/or analyzed during the current study are available from the corresponding author on reasonable request.

## Funding

This study was funded by the 10.13039/501100001809National Natural Science Foundation of China (81970763) and 10.13039/100025989CAMS Innovation Fund for Medical Sciences (CIFMS, 2017-I2M-4-003).

## Conflict of interest

The authors declare that they have no conflicts of interest to disclose.
